# Invasive pulmonary aspergillosis in immunocompetent patients hospitalised with influenza A-related pneumonia: a multicenter retrospective study

**DOI:** 10.1186/s12890-020-01257-w

**Published:** 2020-09-09

**Authors:** Liang Chen, Xiudi Han, Yanli Li, Chunxiao Zhang, Xiqian Xing

**Affiliations:** 1 Department of Infectious Diseases, Beijing Jishuitan Hospital, 4th Medical College of Peking University, Beijing, China; 2 Department of Pulmonary and Critical Care Medicine, Qingdao Municipal Hospital, Qingdao City, Shandong Province China; 3 Department of Infectious Diseases and Clinical Microbiology, Beijing Chao-Yang Hospital, Capital Medical University, Beijing, China; 4 Department of Pulmonary and Critical Care Medicine, Beijing Huimin Hospital, Beijing, China; 5 Department of Pulmonary and Critical Care Medicine, the 2nd People’s Hospital of Yunnan Province, Kunming City, Yunnan Province China

**Keywords:** Influenza A, Pneumonia, Invasive pulmonary aspergillosis, Risk factor

## Abstract

**Background:**

Increasing cases of pulmonary aspergillosis (IPA) in immunocompetent patients with severe influenza have been reported. Howevere, the risk factors for occurence and death are largely unknown.

**Methods:**

Data of hospitalised patients with influenza A-related pneumonia (FluA-p) obtained from five teaching hospitals from 2031 to 2018, were reviewed. Univariate and multivariate logistical regression analyses were performed to determine the risk factors involved in the acquisition and 60-day mortality in IPA patients.

**Results:**

Of the 693 FluA-p patients included in the study, 3.0% (21/693) were IPA patients with a 60-day mortality of 42.9% (9/21). Adjusted for confounders, a Cox proportional hazard model showed that IPA was associated with increased risk for 60-day mortality [hazard ratio (*HR)* 4.336, *95% confidence interval (CI)* 1.191–15.784, *p* = 0.026] in FluA-p patients. A multivariate logistic regression model confirmed that age (odd ratio (*OR)* 1.147, *95% CI* 1.048–1.225, *p* = 0.003), systemic corticosteroids use before IPA diagnosis (*OR* 33.773, *95% CI* 5.681–76.764, *p* <  0.001), leukocytes > 10 × 10^9^/L (*OR* 1.988, *95% CI* 1.028–6.454, *p* = 0.029) and lymphocytes < 0.8 × 10^9^/L on admission (*OR* 34.813, *95% CI* 1.676–73.006, *p* = 0.022), were related with the acquisition of IPA. Early neuraminidase inhibitor use (*OR* 0.290, *95% CI* 0.002–0.584, *p* = 0.021) was associated with a decreased risk for a 60-day mortality in IPA patients.

**Conclusions:**

Our results showed that IPA worsen the clinical outcomes of FluA-p patients. The risk factors for the acquisition and death were helpful for the clinicians in preventing and treating IPA.

## Background

Influenza is a respiratory infectious disease, caused by influenza viruses, and which can present seasonal epidemics and pandemics [1, 2]. Despite the progress in medical technologies and economic development, influenza still causes considerate complications and mortality [3]. Following infection by influenza viruses, patients can show a broad spectrum of clinical symptoms, ranging from self-limited upper respiratory tract illness to severe pneumonia and acute respiratory distress syndrome (ARDS) [4]. It was estimated that every year, 10–20% of the global population experienced symptomatic influenza, including 3–5 million severe illnesses and 260–640 thousand deaths [5].

Nearly half of severe influenza patients present with pneumonia, which is mostly caused by influenza A [6]. Influenza pneumonia is often coinfected with other pathogens and this worsen the clinical symptoms and deteriorates the outcomes [7, 8]. Previous studies found, that *Streptococcus pneumoniae, Staphylococcus aureus* and *Haemophilus influenzae*, were the most common etiologies in coinfected influenza [9]. The Chinese and American guidelines recommend empiric antibiotics use to fight the pathogens associated with severe influenza [10, 11]. Invasive pulmonary aspergillosis mostly and traditionally occurs in immunocompromised hosts, such as patients with hematopoietic stem cell transplantation, granulocyte deficiency and organ transplant recipients; but rarely in those with normal immune function [12, 13]. In recent years, more cases of IPA have been reported in severe influenza patients and with increased mortality [14–16]. The most notable was that over 30% of these cases had no classic immunocompromised factors.

However, there are limited data on influenza-associated pulmonary aspergillosis (IAPA), especially in prior immunocompetent patients. The incidence and disease characteristics were largely unknown. Identifying those patients with high risk for IPA, remains challenging. We carried out this multicenter retrospective study with the following purposes: i) to investigate the incidence and risk factors for IPA acquisition in immunocompetent, adult patients hospitalised with influenza A-related pneumonia (FluA-p); ii) to explore the risk factors associated with a 60-day mortality in IAPA patients.

## Methodology

### Study design and population

We screened hospitalised patients for positive influenza A virus RNA using respiratory specimens by reverse transcription polymerase chain reaction (RT-PCR) from microbiology laboratories of five teaching hospitals in Beijing, Shandong and Yunnan Provinces and during the period from 1st January to 31st December 2018 (the details of the five centers are shown in Additional file 1: Appendix file 1). From this data, we retrieved all cases had both influenza and radiograph proven pneumonia.

Patients were excluded if [17] (1) age < 18 years; (2) pneumonia onset ≥48 h after admission and not been hospitalised within the last 28 days, because the consensus of nosocomial pneumonia caused by influenza was difficult; (3) Immunocompromised status [18].

### Group division

We divided the patients into two groups: the case group included patients with FluA-p, who subsequently became infected with proven and/or probable invasive pulmonary aspergillosis (IPA group); and the control group that included patients with FluA-p and who showed no evidence of *Aspergillus* infection while hospitalised.

### Study definitions

Patients with FluA-p were defined as patients with respiratory symptoms and a new pulmonary infiltrate on the chest radiograph, combined with positive influenza virus A RT-PCR testing during the influenza seasons.

A Proven IPA was defined as the microscopic evidence of dichotomous branching hyphae with a positive culture for *Aspergillus* through an endobronchial biopsy, irrespective of host factors or clinical features [18].

According to the revised definitions of invasive fungal diseases from the European Organization for Research and Treatment of Cancer/Invasive Fungal Infections Cooperative Group and the National Institute of Allergy and Infectious Diseases Mycoses Study Group (EORTC/MSG) in 2019 [18], a probable IPA required a host factor, clinical features and mycological evidence of aspergillosis. However, these criteria were created for immunosuppressed hosts and influenza-related aspergillosis may occur in previously normal hosts. Thus, the host factors were not required in our study. The clinical features included one of the following signs or symptoms: refractory fever to at least 3 days of appropriate antibiotic treatment; recrudescent fever after a period of defervescence of at least 48 h, while still on antibiotics and without other apparent cause; dyspnea; hemoptysis; pleural friction rub or chest pain; worsening respiratory insufficiency in spite of appropriate antibiotic therapy and ventilatory support. The radiological criteria included any infiltrates on pulmonary imaging detected by chest x-ray or CT scan of the lungs. The mycological evidence included: a positive *Aspergillus* culture from a bronchoalveolar lavage (BAL); a galactomannan (GM) optical index on BAL of ≥1.0; a GM optical index on serum of ≥0.5 [18].

Early neuraminidase inhibitor (NAI) treatment was defined as any NAI (e.g., oseltamivir, zanamivir and peramivir) administered within 2 days after disease onset [10, 11]. The coinfection with other *Aspergillus* pathogens was defined by community-acquired respiratory co-pathogens that was identified within 2 days of hospital admission [19]. The conditions of a community-acquired respiratory co-pathogen was defined as the definite or probable etiology (Additional file 1: Appendix 2). Immunocompetent hosts were defined as patients without immunocompromised factors described above.

### Data collection

Data was retrospectively collected and included demographic information, underlying disease (Additional file 1: Appendix 3), clinical manifestations, laboratory and radiological findings, microbiologic diagnosis, treatment (use of antiviral agents, corticosteroids, vasopressors and mechanical ventilation), clinical outcomes (complications during hospitalisation, admittance to the intensive care unit (ICU) and a 60-day mortality).

### Statistical analysis

All data were analyzed with SPSS 22.0 and measurement data were tested for normality by Kolmogorov-Smirnov. The measurement data of normal distributions were reported as mean ± standard deviation. Measurements data of non-normal distributions were reported as median. The categorical variables were analyzed by the Chi-square test or Fisher’s exact test, and continuous variables were analyzed by the student t-test or the Mann–Whitney U-test. A *p*-value of < 0.05 was considered statistically significant and all probabilities were two-tailed. A Cox proportional hazard model was performed to evaluate the effect of IPA on a 60-day mortality in FluA-p patients. The model was adjusted by age, gender, comorbidities, leukocytes > 10 × 10^9^/L, serum procalcitonin > 0.1 ng/ml, coinfection with non-*Aspergillus* pathogens and early use of neuraminidase inhibitors. The clinical characteristics were compared between patients in the IPA and control groups (Table 1). Variables with *p*-values of ≤0.05 on univariate analysis were subsequently entered into the backward stepwise logistic regression analysis to identify risk factors for the acquisition and death in IPA patients.

**Table 1:** Demographic and clinical characteristics between the two groups

Variables	Total (***n*** = 693)	IPA group (***n*** = 21)	Control group (***n*** = 672)	***p-***value^**a**^
Age (years, median, IQR)	61.0 (36.0–76.0)	67.0 (61.0–82.0)	60.0 (36.0–76.0)	**< 0.001**
Male (*n*, %)	461 (66.5)	18 (85.7)	443 (65.9)	0.058
BMI (kg/m^2^, mean ± SD)	24.4 ± 3.6	21.5 ± 0.4	24.5 ± 3.6	**0.014**
Comorbidities (*n*, %)	402 (58.0)	18 (85.7)	384 (57.1)	**0.009**
Diabetes mellitus	92 (13.3)	15 (71.4)	77 (11.5)	**< 0.001**
Cerebrovascular disease	72 (10.4)	4 (19.0)	68 (10.1)	0.338
COPD	40 (5.8)	6 (28.6)	34 (5.1)	**< 0.001**
Asthma	19 (2.7)	3 (14.3)	16 (2.4)	**0.009**
Chronic kidney disease	16 (2.3)	2 (9.5)	14 (2.1)	0.134
Malignant solid tumor	16 (2.3)	0 (0.0)	16 (2.4)	> 0.999
Chronic congestive heart failure	3 (0.4)	3 (14.3)	0 (0.0)	**< 0.001**
Smoking history (*n*, %)	243 (35.1)	7 (33.3)	236 (35.1)	0.866
Alcoholism history (*n*, %)	92 (13.3)	0 (0.0)	92 (13.7)	0.135
Antibiotics use before admission (*n*, %)	587 (84.7)	15 (71.4)	572 (85.1)	0.159
Clinical characteristics (*n*, %)				
Confusion	32 (4.6)	0 (0.0)	32 (4.8)	0.620
SBP < 90 mmHg	8 (1.2)	1 (4.8)	7 (1.0)	0.593
Leukocytes > 10 × 10^9^/L	118 (17.0)	15 (71.4)	103 (15.3)	**< 0.001**
Lymphocytes < 0.8 × 10^9^/L	299/677 (44.2)	15 (71.4)	284/656 (43.3)	**0.011**
HB < 100 g/L	69 (10.0)	5 (23.8)	64 (9.5)	0.075
Albumin < 35 g/L	58/639 (9.1)	6 (28.6)	52/618 (8.4)	**0.006**
BG > 11 mmol/L	48 (6.9)	0 (0.0)	48 (7.1)	0.405
BUN > 7 mmol/L	183/685 (26.7)	9 (42.9)	174/664 (26.2)	0.090
Serum PCT > 0.1 ng/ml	248/541 (45.8)	2 (9.5)	246/520 (47.3)	**0.001**
PO_2_/FiO_2_ < 300 mmHg	340/639 (53.2)	16 (76.2)	324/618 (52.4)	**0.032**
Radiology (*n*, %)				
Cavity	19 (2.7)	3 (14.3)	16 (2.4)	**0.009**
Multiple nodules	151 (21.8)	8 (38.1)	143 (21.3)	0.116
ICs use before IPA diagnosis (*n*, %)	3 (0.4)	3 (14.3)	0 (0.0)	**< 0.001**
Systemic corticosteroids use before IPA diagnosis (*n*, %)	132 (19.0)	18 (85.7)	114 (17.0)	**< 0.001**
Dose of systemic corticosteroids ^a^ (mg/kg, mean ± SD)	0.6 ± 0.3	0.6 ± 0.3	0.6 ± 0.3	0.853
Early NAIs ^b^ use (*n*, %)	232 (33.3)	10 (47.6)	222 (33.0)	0.163
Coinfection with other community-acquired pathogens (*n*, %)	265 (38.2)	3 (14.3)	262 (39.0)	**0.016**

*IQR* interquartile range, *SD* standard deviation, *BMI* body mass index, *COPD* chronic obstructive pulmonary disease, *SBP* systolic blood pressure, *RR* respiratory rates, *WBC* white blood cell count, *HB* hemoglobin, *BG* blood glucose, *BUN* blood urea nitrogen, *PCT* procalcitonin, *PO*_*2*_*/FiO*_*2*_ arterial pressure of oxygen/fraction of inspiration oxygen, *ICs* inhaled corticosteroids, *NAIs* neuraminidase inhibitors; a: methylprednisolone or its equivalent; b: Neuraminidase inhibitors refer to any dose of oseltamivir, zanamivir, and peramivir; ^**a**^: IPA group vs control group. The bolded values are p-values < 0.05, which represent significant differences between subgroups

## Results

### Screening process

**Fig. 1:**
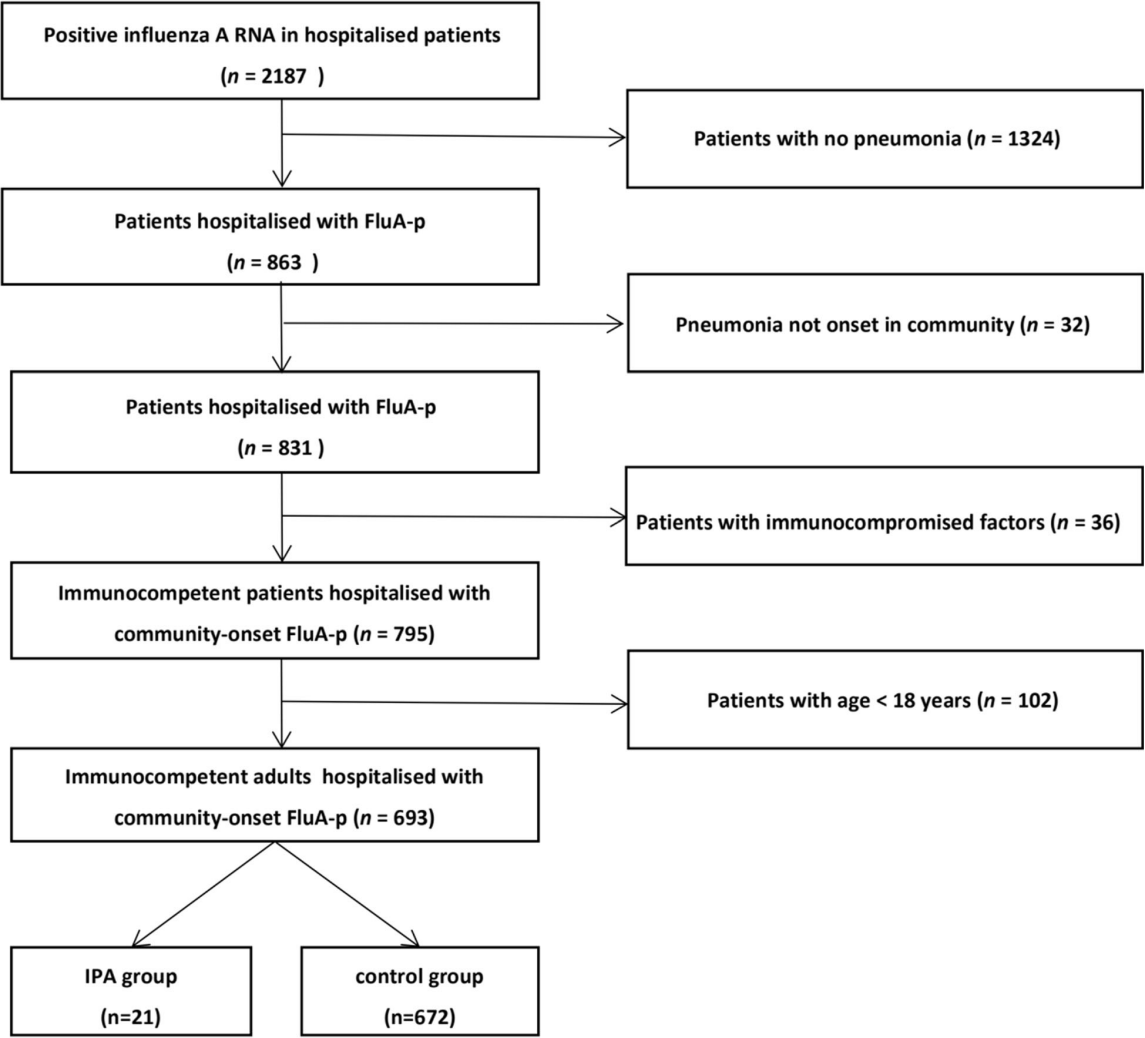
Patient screening algorithm for FluA-p

We screened 2187 hospitalised patients with positive influenza A RNA. Overall, 693 immunocompetent adult patients hospitalised with FluA-p onset in the community were entered into the final analysis. The proportion of patients who developed IPA during hospitalisation was 3.0% (21/693) (Fig. 1).

### Overview of patients with FluA-p

Overall, the median age was 61.0 years old and the male accounted for 66.5% (461/693). Fifty-eight percent of patients (402/693) had at least one underlying disease with the top three being diabetes mellitus 13.3% (92/693), cerebrovascular disease 10.4% (72/693) and chronic obstructive pulmonary disease 5.8% (40/693). The proportion of patients with PO_2_/FiO_2_ < 300 mmHg was 53.2% (340/639). Cavities and multiple nodules in chest radiology were seen in 2.7% (19/693) and 21.8% (151/693) of patients, respectively.

Nineteen percent (132/693) of FluA-p patients used a systemic dose of 0.6 ± 0.3 mg/kg corticosteroids before IPA diagnosis. All patients were administrated with NAI during the disease course, while 33.3% (231/693) received NAIs within the 48 h after illness onset. 24.1% (167/693) of patients had complications with respiratory failure, 21.2% (147/693) had heart failure, 5.2% (36/693) had septic shocks during hospitalisation, 26.3% (182/693) of patients were admitted to intensive care unit (ICU) and the 60-day mortality was 20.9% (145/693) (Table 1).

### Comparisons between the IPA and control patients

Compared with control patients, the IPA patients were older (67.0 yrs. vs. 60.0 yrs., *p* <  0.001), had more frequency of diabetic (71.4% vs. 11.5%, *p* <  0.001), chronic pulmonary disease (28.6% vs 5.1%, *p* <  0.001), asthma (14.3% vs. 2.4%, *p* = 0.009) and chronic heart failure (14.3% vs. 0.0%, *p* <  0.001), and lower levels of body mass index (BMI) [(21.5 ± 0.4) kg/m^2^ vs. (24.5 ± 3.6) kg/m^2^, *p* = 0.014]. The proportion of leukocytes > 10 × 10^9^/L (71.4% vs. 15.3%, *p* <  0.001), lymphocytes < 0.8 × 10^9^/L (71.4% vs. 43.3%, *p* = 0.011), albumin < 35 g/L (28.6% vs. 8.4%, *p* = 0.006), PO_2_/FiO_2_ < 300 mmHg (76.2% vs 52.4%, *p* = 0.032) and radiologic cavities (14.3% vs. 2.4%, *p* = 0.009), were significantly higher in the IPA patients; while, serum procalcitonin (PCT) >  0.1 ng/ml (9.5% vs. 47.3%, *p* = 0.001) was more common in the control patients. More IPA patients used corticosteroids inhalers (14.3% vs. 0.0%, *p* <  0.001) and systemic corticosteroids (85.7% vs. 17.0%, *p* <  0.001) before IPA diagnosis. However, no significant differences in the dose of systemic corticosteroids was observed.

Complications of respiratory failure (100.0% vs. 21.7%, *p* <  0.001), heart failure (42.9% vs. 20.5%, *p* = 0.028) and septic shock (85.7% vs. 2.7%, *p* <  0.001) were more frequent in IPA patients. The proportion of patients needing noninvasive ventilation (42.9% vs. 22.3%, *p* = 0.014), invasive ventilation (85.7% vs. 20.8%, *p* <  0.001) and vasopressor use (52.4% vs. 2.4%, *p* <  0.001), were higher in IPA patients. More IPA patients were admitted to ICU (71.4% vs. 24.9%, *p* <  0.001) and had a higher 60-day mortality rate (42.9% vs. 18.9%, *p* = 0.015) (Table 2).

**Table 2:** Supportive treatments and clinical outcomes between the two groups

Variables	Total (***n*** = 693)	IPA group (***n*** = 21)	Control group (***n*** = 672)	***p***-value^**a**^
Vasopressor use (*n*, %)	27 (3.9)	11 (52.4)	16 (2.4)	**< 0.001**
Length of vasopressor use (days, median, IQR)	1.0 (0.5–3.0)	2.0 (0.5–4.5)	1.5 (1.0–2.0)	0.185
Noninvasive ventilation (*n*, %)	159 (22.9)	9 (42.9)	150 (22.3)	**0.014**
Length of noninvasive ventilation (days, median, IQR)	4.0 (1.0–8.0)	2.0 (2.0–10.0)	5.0 (1.0–8.0)	**0.009**
Invasive ventilation (*n*, %)	158 (22.8)	18 (85.7)	140 (20.8)	**< 0.001**
Length of invasive ventilation (days, median, IQR)	4.0 (1.0–9.0)	9.0 (7.0–11.0)	4.0 (1.0–9.0)	**0.003**
Complications during hospitalisation (*n*, %)				
Respiratory failure	167 (24.1)	21 (100.0)	146 (21.7)	**< 0.001**
Heart failure	147 (21.2)	9 (42.9)	138 (20.5)	**0.028**
Septic shock	36 (5.2)	18 (85.7)	18 (2.7)	**< 0.001**
Acute kidney failure	27 (3.9)	3 (14.3)	24 (3.6)	0.054
Bloodstream infection	8 (1.2)	0 (0.0)	8 (1.2)	> 0.999
Admittance to ICU (*n*, %)	176 (26.3)	15 (71.4)	161 (24.0)	**< 0.001**
Length of stay in ICU (days, median, IQR)	8.0 (6.0–13.0)	9.0 (7.0–11.0)	8.0 (6.0–13.0)	0.473
LOS (days, median, IQR)	10.0 (8.0–14.0)	24.0 (11.0–42.0)	10.0 (7.0–13.0)	**< 0.001**
60-day mortality (*n*, %)	136 (19.6)	9 (42.9)	127 (18.9)	**0.015**

*LOS* length of stay in hospital, *ICU* intensive care unit; ^**a**^: IPA group vs control group. The bolded values are p-values < 0.05, which represent significant differences between subgroups

### Diagnosis of IPA

The mean duration from the diagnosis of IPA to the day of admission was 6.4 ± 4.8 days, with a range of 2–18 days. A serum GM test was performed in 15 of the 21 IPA patients. Seventeen IPA patients were subjected to a GM test in BAL. Fourteen patients had a GM optical index on BAL of ≥1.0, five patients a GM optical index of single serum of ≥0.5.

In all 21 IPA patients, a BAL culture was performed that led to the identification of *Aspergillus* in 6 patients’ cultures. Two patients were diagnosed as proven IPA by trans-bronchial lung biopsy (both were *Aspergillus fumigatus*). A probable IPA diagnosis was performed in 19 of the 21 IPA patients (Table 3).

**Table 3:** Diagnosis of IPA

Variables	IPA group (***n*** = 21)	Control group (***n*** = 672)
Serum GM test (*n*, %)	15 (71.4)	322 (47.9)
BAL GM test (*n*, %)	17 (81.0)	167 (24.9)
Serum GM ≥ 0.5	5 (4.8)	0 (0.0)
BAL GM ≥ 1.0	14 (66.7)	0 (0.0)
BAL *Aspergillus* culture (*n*, %)	21 (100.0)	146 (21.7)
Positive	6 (28.6)	0 (0.0)
Lung tissue microscopy (*n*, %)	4 (19.0)	18 (2.7)
Positive	2 (9.5)	0 (0.0)
Proven IPA (*n*, %)	2 (9.5)	0 (0.0)
Probable IPA (*n*, %)	19 (90.5)	0 (0.0)

*GM* galactomannan, *BAL* bronchoalveolar lavage

### Coinfection with non-*Aspergillus* pathogens isolated in FluA-p patients

Coinfection with other community-acquired pathogens was diagnosed in only 3 of 21 IPA patients and 1 patient was diagnosed with *S. pneumoniae,* 1 patient with *P. aeruginosa* and 1 with *K. pneumoniae*. While, 39.0% (262/672) of control patients were diagnosed with non-*Aspergillus* etiologies, *S. pneumoniae* was the most common diagnosed pathogen with 33.2% (87/262), followed by *K. pneumoniae* 30.5% (80/262) and *Staphylococcus aureus* 20.6% (54/262) (Additional file 1: Appendix file 4).

### Effect of IPA on the 60-day mortality of FluA-p patients

Adjusted for age, gender, comorbidities, blood leukocyte counts > 10 × 10^9^/L, serum PCT >  0.1 ng/ml, coinfection with other pathogens and early NAIs use, a Cox proportional hazard model showed that IPA was associated with an increased risk in the 60-day mortality of FluA-p patients [hazard ratio (*HR)* 4.336, *95% confidence interval (CI)* 1.191–15.784, *p* = 0.026) (Table 4).

**Table 4:** The impact of IPA on the 60-day mortality in FluA-p patients

Variable	Univariate Cox regression		Multivariate Cox regression	
	***HR (95% CI)***	p***-***value	^**a**^adjusted ***HR (95%CI)***	p***-***value
IPA	3.219 (1.328–7.803)	0.010	4.336 (1.191–15.784)	0.026

*HR* hazard ratio, *CI* interval confidence

^**a**^adjusted by age, gender, comorbidities(chronic pulmonary disease, cerebrovascular disease, asthma, diabetes mellitus, chronic kidney disease, malignant solid tumor, chronic congestive heart failure), leukocytes > 10 × 10^9^/L, serum procalcitonin > 0.1 ng/ml, coinfection with non-*Aspergillus* other pathogens, early NAIs use

**Fig. 2:**
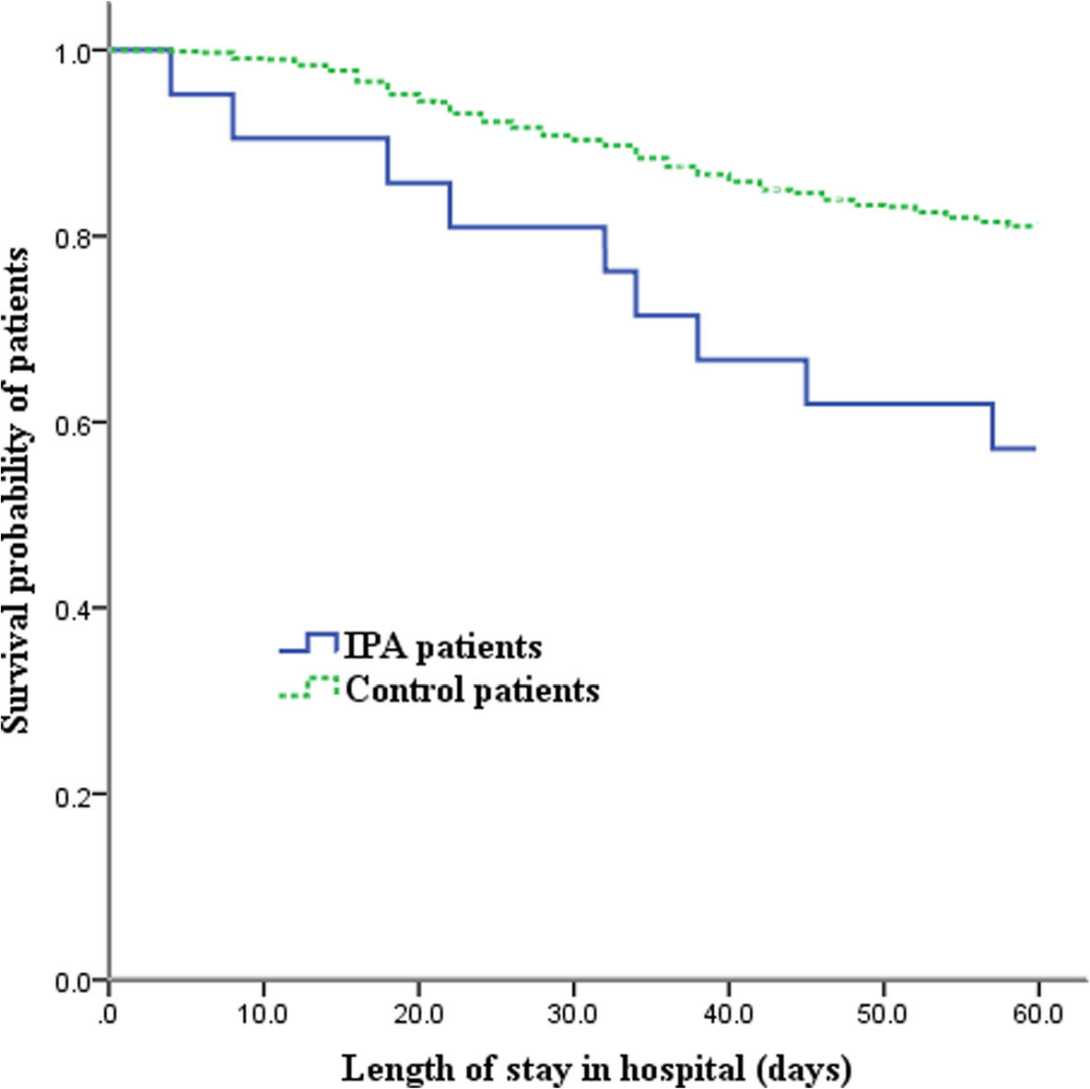
Kaplan-Meier survival graph for FluA-p patients with and without IPA (censored at 60d)

Kaplan-Meier survival curve showed that the 60-day mortality of the IPA patients was significantly higher than that of the control patients (*p* = 0.006 for the log rank test) (Fig. 2).

### Risk factors associated with IPA occurrence in FluA-p patients

To explore the risk factors for IPA acquisition, the following variables were entered into the backstep logistic regression model: age, BMI, diabetes mellitus, asthma, chronic congestive heart failure, leukocytes > 10 × 10^9^/L, lymphocytes < 0.8 × 10^9^/L, albumin < 35 g/L, serum PCT >  0.1 ng/ml, cavity on chest radiology, use of inhaled corticosteroids and systemic corticosteroids before IPA diagnosis, and coinfection with other community-acquired pathogens, and the analyses led to the following results: age (*OR* 1.147, *95% CI* 1.048–1.225, *p* = 0.003), systemic corticosteroids use before IPA diagnosis (*OR* 33.773, *95% CI* 5.681–76.764, *p* <  0.001), leukocytes > 10 × 10^9^/L (*OR* 1.988, *95% CI* 1.028–6.454, *p* = 0.029) and lymphocytes < 0.8 × 10^9^/L on admission (*OR* 34.813, *95% CI* 1.676–73.006, *p* = 0.022). These results were proven to be independently related to the IPA acquisition in FluA-p patients (Table 5).

**Table 5:** Predictors for acquisition and 60-day mortality of IPA in FluA-p patients

Predictors for IPA acquisition	p***-***value	***OR (95% CI)***
Age	0.003	1.147 (1.048–1.225)
Systemic corticosteroids use before IPA diagnosis	< 0.001	33.773 (5.681–76.764)
Leukocytes > 10 × 10^9^/L	0.029	1.988 (1.028–6.454)
Lymphocyts < 0.8 × 10^9^/L	0.022	34.813 (1.676–73.006)
**Predictors for 60-day mortality of IPA patients**		
Early NAIs use	0.021	0.290 (0.002–0.584)

*OR* odd ratio

### Predictors for a 60-day mortality of IPA in FluA-p patients

The demographic features and comorbidities were similar between the survival and deceased patients with IPA. Of the 21 patients that received an antifungal treatment within 24 h after IPA diagnosis, 18 patients used voriconazole and 3 patients used a combination treatment (voriconazole + echinocandin). However, no significant difference was found in antifungal therapy between the two groups. Compared with the survival group, the deceased patients’ group had a higher proportion of lymphocytes < 0.8 × 10^9^/L (100.0% vs 50.0%, *p* = 0.043) and lower proportion of early NAIs use (11.1% vs 75.0%, *p* = 0.014) (Additional file 1: Appendix file 5).

A multivariate logistic regression model confirmed early NAIs use (*OR* 0.290, *95% CI* 0.002–0.584, *p* = 0.021) and that was the only predictor for the 60-day mortality in IPA patients (Table 5).

## Discussion

Our study has two important findings: 1) the prevalence of IPA in immunocompetent adult patients hospitalised with FluA-p, was 3.0%. However, it was associated with increased mortality; 2) we identified age, leukocytes, lymphocytes and systemic corticosteroids use as risk factors for IPA diagnosis. Early NAIs use was related to better outcomes, which were helpful in the prevention and treatment of IPA patients with severe influenza.

There are rare data on the incidence of IPA among all hospitalised FluA-p patients and previous studies were limited to patients admitted to ICU. In our study, the incidence of IPA in ICU patients was 8.2%, which was consistent with the 7.2–8.8% reported by Rice [20] and Martin-Loeches [21]; but, this was lower than the reported 19.2% in the Schauwvlieghe’s study [22]. Further analysis revealed that all IPA patients had respiratory failure complications and an IPA incidence of 12.6%. This observation is very close to that of the Schauwvlieghe’s study, in which the IPA incidence was 14.2% in non-immunocompromised severe influenza patients. The difference in reported IPA incidences could be explained by the severity of influenza illness, the detection capability of IPA and the discrepancy in ICU admission criteria in the different medical systems.

Although the IPA incidence in our study was not high, the 60-day mortality was as high as 42.9%, which was in accordance with previous reports of 33–71% [12, 14–16, 19, 22–24]. In addition to the genetic background, there were at least two reasons for the difference in mortality among those studies: 1) the influence of the patients’ immunity status before influenza onset. In the Schauwvlieghe’s study [22], the 90-day mortality in immunocompetent patients with IPA, was 33%; while, it was 71% in patients with immunocompromised factors. Among Huang’s research population [24], 24% received immunosuppressive agents and the overall ICU mortality was 41.3%. All the patients in our study were previously immunocompetent and the mortality was lower than that in the previously reported immunocompromised patients’ studies. 2) the outcomes were profoundly affected by the awareness, timing and approach of pathogenic testing that were performed for invasive aspergillosis, leading to a delayed IPA diagnosis and treatment. For example, a center in the Schauwvlieghe’s [22] study paid more attention to IPA because of several previous reports. As a result, the diagnostic and survival rate of IPA were much higher than that in other centers. Adjusted by age, sex, comorbidities, early use of NAIs and probable co-infection with other etiologies, a Cox proportional hazard model showed, that IPA independently increases the risk for 60-day mortality in FluA-p patients, by 3 times. Our results implied the importance of IPA screening in patients with severe influenza pneumonia, regardless of their previous immunity status. This allows an early diagnosis in patients, that prompts carrying out an antifungal treatment, as soon as possible.

In our study, IPA patients were older in age and with frequent comorbidities. Diabetes and chronic airway diseases (such as COPD and asthma) were the most common underlying diseases. Hypoproteinemia was common, suggesting that their nutritional statuses were poor. When the radiological findings of IPA patients were reviewed, it showed mainly pulmonary infiltrates, with a slightly higher proportion of cavities than the control patients; but, no obvious halo sign was found. Previous studies also showed that the halo sign was only seen in less than 5% of patients with secondary IPA influenza [25, 26]. The multivariate logistic regression analysis confirmed, that age, increased leukocyte counts, decreased lymphocyte counts and systemic corticosteroids use, were independent risk factors for the acquisition of IPA in immunocompetent patients hospitalised with FluA-p. It is believed that the pathogenesis of invasive aspergillosis, in the setting of influenza infection, may be due to both local and systemic effects of the virus. Local effects include influenza and inflammation damage of the bronchoalveolar epithelial cells, that lead to the impairment of the barrier function and dysfunction of ciliary motility and clearance [27]. Systemically, influenza alters the Th1/Th2 balance and causes lymphopenia. The immune function of the elderly patients significantly decreased, and their resistance to infection was poor. Previous studies also showed that elderly influenza patients, were more vulnerable to secondary infections [28]. Elevated leukocyte counts in influenza pneumonia patients is usually associated with bacterial or fungal coinfection; while, serum PCT is a relatively specific biomarker of bacterial infection [29]. In our study, more than 90% of IPA patients had a serum PCT of < 0.1 ng/ml, indicating that the coinfection with bacteria was not common. Therefore, the elevated leukocytes with normal serum PCT level, strongly suggested fungal infection. Lymphocytes reflected the function of cellar immunity, which was the main anti-viral mechanism in humans. The suppression of cellar immunity delays the clearance of the virus, along with a more serious damage of airway, thus creating conditions for an invasive *Aspergillus* infection.

The steroids are the most common immunomodulators in clinic, with powerful depressive effects on both cellar immunity and humoral immunity. Corticosteroids use often causes secondary fungal infection [30]. Our study confirmed the association of systemic corticosteroids use and occurrence of IPA as previously reported [22, 24]. Meanwhile, we found, that even a low-to-moderate dose and short-term systemic corticosteroids use, would increase the risk for of IPA acquisition in the predisposition to immune suppression, caused by severe influenza. Traditionally, it was believed that a prolonged use of steroids (at least 3 weeks and a prednisone equivalent of > 0.3 mg/kg/d) was related to IPA occurrence [31]. It should be noted that there may be overuse of systemic steroids in influenza patients. In numerous reports, more than 50% of influenza patients received systemic corticosteroids, which were proven to be associated with poor outcomes [32, 33]. In our study, 19% of FluA-p patients received systemic steroids during hospitalisation. Although in severe influenza, uncontrolled immune response is involved in organic damage and increased mortality. In animal models, corticosteroid treatment was found to decrease mortality and ameliorate acute lung injury induced by influenza [34]. By now, except for septic shock patients with vasopressor-dependence or adrenal insufficiency, there is no consensus on steroids use in the treatment of severe influenza. Our study confirmed, that the systemic steroids use, was associated with increased risk for IPA and with an unacceptable high mortality, and even in prior immunocompetent FluA-p patients. Therefore, we thought that it was urgent to regulate the use of systemic corticosteroids in the influenza setting.

In our study, early NAIs use was proven to be the only predictor associated with better outcomes in IAPA patients, by decreasing the risk of more than 70% for a 60-day mortality. Previous studies also proved that the sooner NAIs were used, the better were the outcomes in severe influenza patients [35, 36]. According to the mechanism of IAPA, it is reasonable that early inhibition of viral duplication and alleviation of lung damage, caused by virus and inflammation, can create favorable conditions for controlling the occurrence and development of IAPA. However, why early use of NAIs had not shown a decrease in the risk for IAPA incidence, was still unclear. Apart from the small size of the population, our study implies that there is a complex pathogenesis for IPA occurrence in severe influenza patients.

As far as we know, this was the only investigation focused on IPA in immunocompetent patients hospitalised with FluA-p. Unlike other studies that were limited to ICU patients, our study included, not only patients in ICU, but also patients from general wards. IPA patients, that were not admitted to ICU in other studies and for some reasons, could be included into our population; which, reduced the selective bias. In addition, the population in our study had no classic immunocompromised factors. The occurrence of IPA in these patients was most likely to be neglected. Therefore, the results of our study provide a great significance for clinicians as it allows to identify patients with increased risk for IPA acquisition and at an early stage, advocating therefore, a prompt prevention and treatment.

There were some limitations specific to our study: 1) Besides the nature of the retrospective study, the relatively small sample size along with some missing data, might limit the accuracy of the results; 2) Though the latest diagnostic criteria of IPA was used, the proportion of the microbiologic examinations for IPA in the control patients, was low. Especially, BAL samples were only performed in less than 25%; while, a serum GM testing was performed in 47% of control patients. Some studies showed, that with the current diagnostic standard of serum GM optical index (OD) >  0.5, nearly half of IPA cases would be under-detected [37, 38]. In addition, the triggers to perform BAL were not clarified due to the restrospective study design, and IPA complicating influenza might develope post-admission. This may have limited the number of true cases found and caused selective bias. Therefore, the actual IPA incidence in our study might be under-detected; 3) more than 1/3 of the patients had not performed influenza subtype testing and other respiratory tract viruses were not routinely detected. Thus, we could not exclude coinfection with other viruses.

## Conclusions

Our study showed that there is approximatively 3.0% of IPA incidence with an increased mortality that was observed even in immunocompetent patients, hospitalised with FluA-p. Additionally, we identified age, elevated leukocytes, reduced lymphocytes, on the day of admission and systemic corticosteroids use, as risk factors for IPA acquisition, and that early NAIs use was a predictor of better outcome. Meanwhile, it is suggested that these results should be confirmed using prospective and large sample studies to further verify these conclusions.

## Supplementary information

**Additional file 1 : Appendix 1:** Details of Participating centers. **Appendix 2** Definition of microbiological criteria of coinfected other pathogens. **Appendix 3** Definition of underlying diseases. **Appendix 4** Coinfection with other pathogens. **Appendix 5** Univariate analysis between the survival group and the deceased group.

## Data Availability

All data generated or analysed during this study are included in this published article and its supplementary information files.

## Abbreviations

Flu-p: Influenza-related pneumonia
IPA: Invasive pulmonary aspergillosis
NAI: Neuraminidase inhibitor
OR: Odds ratio
HR: Hazard ratio
95% *IC*: 95% Interval confidence
FluA-p: Influenza A-related pneumonia
RT-PCR: Reverse transcription polymerase chain reaction
EORTC/MSG: European Organization for Research and Treatment of Cancer/Invasive Fungal Infections Cooperative Group and the National Institute of Allergy and Infectious Diseases Mycoses Study Group
BAL: Bronchoalveolar lavage
GM: Galactomannan
IQR: Interquartile range
BMI: Body mass index
COPD: Chronic obstructive pulmonary disease
SBP: Systolic blood pressure
Hb: Hemoglobin
BG: Blood glucose
ALB: Albumin
BUN: Blood urea nitrogen
PH: Hydrogen ion index
pO_2_/FiO_2_: Arterial pressure of oxygen/fraction of inspiration oxygen
ICs: Inhaled corticosteroid
PCT: Procalcitonin
ICU: Intensive care unit
