# Supplementary material for: Invasive pulmonary aspergillosis in immunocompetent patients hospitalised with influenza A-related pneumonia: a multicenter retrospective study
Source: BMC Pulm Med. 2020 Sep 9;20:239. doi: 10.1186/s12890-020-01257-w (PMC7479745; doi:10.1186/s12890-020-01257-w)
Supplement: Supplementary file 1 — Additional file 1 : Appendix 1: Details of Participating centers. Appendix 2 Definition of microbiological criteria of coinfected other pathogens. Appendix 3 Definition of underlying diseases. Appendix 4 Coinfection with other pathogens. Appendix 5 Univariate analysis between the survival group and the deceased group. [file 12890_2020_1257_MOESM1_ESM.docx]

**Appendix 1: Details of Participating centers**

| **Name of the hospital** | **Province, city** | **Teaching Hospital** | **Beds** | **Staffs of Clinical Microbioloy Lab** |
| --- | --- | --- | --- | --- |
| Beijing Jishuitan Hospital,  4^th^ Medical College of Peking University | Beijing | Yes | 1500 | 10 |
| Beijing Chao-Yang Hospital **Affiliated to** Capital Medical University | Beijing | Yes | 1400 | 11 |
| **the 2nd People’s Hospital of Yunnan Province** | **Kunming,**  **Yan’an** | Yes | 1302 | 4 |
| **Qingdao Municipal Hospital** | ShanDong,  Qingdao | Yes | 1200 | 4 |
| Beijing Huimin Hospital | Beijing | Yes | 1000 | 2 |

**Appendix 2 Definition of microbiological criteria of coinfected other pathogens**

**Definite**, if one of the following criteria was met:

1. Positive urinary antigen for *Legionella pneumophila* ;
2. Positive urinary antigen for *Streptococcus pneumoniae*;
3. Positive bacterial culture from blood or plural fluid except for coagulase negative *Staphylococcus spp*.
4. Paired sera with a fourfold or more increase in the titers of antibodies to *Mycoplasma pneumoniae* (MP), *Chlamydia pneumonia*, *L pneumophila or* respiratory viruses (Adenovirus, Respiratory syncytial virus)*.* Or Serum IgM antibody (MIF) ≥ 1:16 for *Chlamydia pneumonia.*

**Probable**, if one of the following criteria was met:

1. Detection of respiratory virus in sputum/bronchoalveolar lavage (BAL)/throat swabs by Realtime-PCR according to manufacturer’s instructions, including respiratory syncytial virus (RSV) types A and B, parainfluenza virus (PIV) types 1, 2, 3 and 4, rhinovirus (HRV), enterovirus (EV), coronavirus (hCoV) types 229E, NL63, OC43 and HKU1, parapneumovirus (hMPV), and adenovirus (AdV), bocavirus;
2. Bacteria isolated form purulent sputum (defined as an adequate quality sputum sample with > 25 leukocytes and < 10 epithelial cells per × 100 magnification field) with compatible findings of Gram staining;
3. Detection of *Mycoplasma pneumoniae* (MP), *Chlamydia pneumonia* or *L pneumophila* in sputum/BAL/throat swabs by Realtime-PCR;
4. Serum IgM antibody positive for *Mycoplasma pneumoniae* (MP), or Serum IgG antibody (MIF) ≥ 1:512 for *Chlamydia pneumonia.*

**Appendix 3 Definition of underlying diseases**

1. Chronic pulmonary disease was defined as: persistent airflow limitation, FEV_1_ / FVC < 70% post bronchodilator;
2. Asthma was defined by the history of respiratory symptoms such as wheeze, cough that varied over time and intensity, together with variable respiratory airway limitation;
3. Chronic congestive heart failure was defined as cardiomegaly and ejection fraction ≤ 40%;
4. Cerebrovascular diseases included transient ischemic attack, cerebral hemorrhage, subarachnoid hemorrhage, cerebral infarction;
5. Diabetes mellitus: included diabetes mellitus type 1 and diabetes mellitus type 2, not included impaired glucose tolerance and impaired fasting glycaemia;
6. Chronic kidney disease included diabetic nephropathy, hypertensive renal damage, chronic glomerulonephritis, chronic pyelonephritis, lupus nephritis, IgA nephropathy, nephrotic syndrome, hereditary kidney disease, etc;
7. Immunocompromised status included: ⅰ) recent history of neutropenia (<0.5 × 10^9^ neutrophils/L [< 500 neutrophils/mm^3^] for >10 days) temporally related to the onset of invasive fungal disease；ⅱ) hematologic malignancya; ⅲ) receipt of an allogeneic stem cell transplant; ⅳ) receipt of a solid organ transplant; Ⅴ) prolonged use of corticosteroids (excluding among patients with allergic bronchopulmonary aspergillosis) at a therapeutic dose of ≥ 0.3 mg/kg corticosteroids for ≥ 3 weeks in the past 60 days; ⅵ) treatment with other recognized T-cell immunosuppressants, such as calcineurin inhibitors, tumor necrosis factor-a blockers, lymphocytespecific monoclonal antibodies, immunosuppressive nucleoside analogues during the past 90 days; Ⅶ) treatment with recognized B-cell immunosuppressants, such as Bruton’s tyrosine kinase inhibitors, eg, ibrutinib; Ⅷ) inherited severe immunodeficiency (such as chronic granulomatous disease, STAT 3 deficiency, or severe combined immunodeficiency); Ⅸ) acute graft-versus-host disease grade III or IV involving the gut, lungs, or liver that is refractory to first-line treatment with steroids.
8. Smoking history defined as: cigarette smokers of 10 cigarettes/d during at least the previous year.
9. Alcoholism history defined as: drinking more than 5 bottles of beer (500ml / bottle) or half a catty liquor once in 2 weeks; or drinking more than 2.5 bottles of beer (500ml / bottle) or 2 ounc of white spirit per day for more than five years.
10. Septic shock was defined as hypotension requiring use of vasopressors to maintain mean blood pressure of 65 mmHg or greater and having a serum lactate level greater than 2 mmol/L persisting after adequate fluid resuscitation.
11. Acute renal failure was defined as a more than threefold rise in serum creatinine concentration compared to baselinewith an increase of at least 4 mg/dL, accompanied by reduction of urine excretion to < 0.3 mL/kg for≥ 24 h or anuria for ≥ 12 h.

**Appendix 4 coinfection with other pathogens**

| **Variables** | **Total**  **(*n* = 693)** | **IPA group**  **(*n* = 21)** | **Control group**  **(*n* = 672)** |
| --- | --- | --- | --- |
| Coinfection | 265 (38.2) | 3 (14.3) | 262 (39.0) |
| Pathogens |  |  |  |
| *Streptococcus pneumoniae* | 88 (33.2) | 1 (0.0) | 87 (33.2) |
| *Klebsiella pneumoniae* | 81 (30.6) | 1(33.3) | 80 (30.5) |
| *Staphylococcus aureus* | 54 (20.4) | 0 (0.0) | 54 (20.6) |
| *Haemophilus influenzae* | 17 (6.4) | 0 (0.0) | 17 (6.5) |
| *Pseudomonas aeruginosa* | 12 (4.5) | 1 (66.7) | 11 (4.2) |
| *Klebsiella acidogens* | 7 (2.6) | 0 (0.0) | 7 (2.7) |
| *Acinetobacter* | 5 (1.9) | 0 (0.0) | 5 (1.9) |
| *Proteus spp.* | 3 (1.1) | 0 (0.0) | 3 (1.1) |
| *Stenotrophomonas maltophilia* | 3 (1.1) | 0 (0.0) | 3 (1.1) |
| *Escherichia coli* | 2 (0.8) | 0 (0.0) | 2 (0.7) |
| *Citrobacter spp.* | 1 (0.4) | 0 (0.0) | 1 (0.4) |

8 patients in the control group were coinfected with ≥ 2 pathogens

**Appendix 5 Univariate analysis between the survival group and the deceased group**

| **Variables** | **Survival group**  **(*n* = 12)** | **Deceased group**  **(*n* = 9)** | **p value** |
| --- | --- | --- | --- |
| Age (years, mean±SD) | 69.3±8.7 | 71.7±11.8 | 0.595 |
| Male (*n*, %) | 9 (75.0) | 9 (100.0) | 0.322 |
| Chronic congestive heart failure (*n*, %) | 3 (25.0) | 0 (0.0) | 0.322 |
| Cerebrovascular disease (*n*, %) | 2 (16.7) | 2 (22.2) | > 0.999 |
| Chronic pulmonary disease (*n*, %) | 4 (33.3) | 2 (22.2) | 0.944 |
| Asthma (*n*, %) | 2 (16.7) | 1 (11.1) | > 0.999 |
| Diebetes mellitus (*n*, %) | 10 (83.3) | 5 (55.6) | 0.365 |
| Chronic kidney disease (*n*, %) | 2 (16.7) | 0 (0.0) | 0.486 |
| Leukocytes > 10×10^9^/L (*n*, %) | 9 (75.0) | 6 (66.7) | > 0.999 |
| Lymphocytes < 0.8×10^9^/L (*n*, %) | 6 (50.0) | 9 (100.0) | **0.043** |
| BUN > 7 mmol/L (n, %) | 4 (33.3) | 5 (55.6) | 0.567 |
| HB < 100 g/L (*n*, %) | 4 (33.3) | 1 (11.1) | 0.160 |
| Albumin < 35 g/L (*n*, %) | 4 (33.3) | 2 (22.2) | 0.944 |
| Serum PCT > 0.1 ng/ml (*n*, %) | 1 (8.3) | 1 (11.1) | > 0.999 |
| PO_2_/FiO_2_ < 300 mmHg (*n*, %) | 8 (66.7) | 7 (77.8) | 0.994 |
| Systemic corticosteroids use  before IPA diagnosis (*n*, %) | 9 (75.0) | 9 (100.0) | 0.322 |
| Early NAIs use (*n*, %) | 9 (75.0) | 1 (11.1) | **0.014** |
| Anti-fungal treatment (*n*, %) |  |  |  |
| voriconazole | 9 (75.0) | 9 (100.0) | 0.322 |
| voriconazole+ caspofungin | 3 (25.0) | 0 (0.0) | 0.126 |
| Invasive ventilation (*n*, %) | 9 (75.0) | 9 (100.0) | 0.322 |
| Septic shock (*n*, %) | 10 (83.3) | 8 (88.9) | > 0.999 |

BUN: blood urea nitrogen; HB: hemoglobin; PCT: procalcitonin; PO_2_/FiO_2:_ arterial pressure of oxygen/fraction of inspiration oxygen; NAIs: neuraminidase inhibitors
